# Liproxstatin-1 Alleviated Ischemia/Reperfusion-Induced Acute Kidney Injury via Inhibiting Ferroptosis

**DOI:** 10.3390/antiox13020182

**Published:** 2024-01-31

**Authors:** Zhiyuan Shi, Yifan Du, Jianzhong Zheng, Wenbin Tang, Qing Liang, Zeyuan Zheng, Bin Liu, Huimin Sun, Kejia Wang, Chen Shao

**Affiliations:** 1 Department of Urology, Xiang’an Hospital of Xiamen University, School of Medicine, Xiamen University, Xiamen 361101, China; 24520200156791@stu.xmu.edu.cn (Z.S.); 24520221154702@stu.xmu.edu.cn (Y.D.); 24520180155766@stu.xmu.edu.cn (J.Z.); 24520191153638@stu.xmu.edu.cn (W.T.); 24520211154474@stu.xmu.edu.cn (Z.Z.); 24520221154718@stu.xmu.edu.cn (B.L.); 2 Fujian Provincial Key Laboratory of Organ and Tissue Regeneration, Xiamen Key Laboratory of Regeneration Medicine, Organ Transplantation Institute of Xiamen University, School of Medicine, Xiamen University, Xiamen 361101, China; liangqing@xmu.edu.cn; 3 Central Laboratory, Xiang’an Hospital of Xiamen University, School of Medicine, Xiamen University, Xiamen 361101, China; hmsun@xah.xmu.edu.cn

**Keywords:** liproxstatin-1, ischemia/reperfusion, acute kidney injury, ferroptosis, EGR1

## Abstract

Ferroptosis, as a novel regulable cell death, is characterized by iron overload, glutathione depletion, and an accumulation of lipid peroxides. Recently, it has been discovered that ferroptosis is involved in ischemia/reperfusion (I/R)-induced acute kidney injury (AKI) and plays a crucial role in renal tubular cell death. In this study, we tried to investigate the effect and mechanism of liproxstatin-1 (Lip-1) in I/R-induced AKI and seek the key regulator of ferroptosis in I/R-induced AKI. Mice were administrated with clamping bilateral renal pedicles for 30 min. We found that early growth response 1 (EGR1) might be a key regulator of ferroptosis, and Lip-1 could suppress ferroptosis via EGR1. Meanwhile, Lip-1 could reduce macrophage recruitment and the release of inflammatory cytokines. These findings indicated that Lip-1 alleviated I/R-induced AKI via regulating EGR1, and it might pave the theoretical basis of a new therapeutic strategy for I/R-induced AKI.

## 1. Introduction

Acute kidney injury (AKI) is a group of clinical syndromes, which leads to the remaining part of the kidney function being unable to keep the electrolyte balance and acid–base balance. It has been estimated that more than 13.3 million patients were affected by AKI and about 1.7 million individuals lose their lives around the world each year [1]. The incidence of AKI in hospitals is about 10~15%, and it could be more than 50% in several departments (such as oncology, transplantation center, intensive care unit (ICU), and cardiac surgery) [2,3,4]. The in-hospital mortality rates of AKI are 5.1%, 13.7%, and 24.8% from stage I to stage III, respectively [5]. AKI is regarded as a crucial risk factor that could progress to chronic kidney disease (CKD) and end stage kidney disease [6]. Moreover, there are nearly 2 million AKI patients that could not make a complete recovery, and the risk of progressing to CKD is high [7]. In addition, the 30-day and 90-day mortality of patients without AKI in ICU were much lower than their counterparts with AKI [8]. It is obvious that patients without AKI possessed better medium-term to long-term outcomes than their counterparts with AKI [9,10]. Many factors could result in the occurrence of AKI, for instance ischemia/reperfusion (I/R), sepsis, folic acid, and cisplatin. The injury and death of renal tubular cells are the early and pivotal pathophysiology of I/R-induced AKI, and they are accompanied by peritubular endothelial dysfunction and inflammatory cell infiltration [11,12].

Ferroptosis was first described by Stockwell in 2012 [13]. It is a novel regulable cell death and characterized by iron overload, glutathione (GSH) depletion, and an accumulation of lipid peroxides. Ferroptosis has been widely explored in various diseases, such as various cancers, neurodegenerative diseases, cardiovascular diseases, and many organ injuries [14]. Many studies have shown that the GSH peroxidase 4 (GPX4) performed a crucial role in repressing ferroptosis and might be a potential target [15]. A large amount of renal tubular cell death occurred in *Gpx4*^−/−^ mice, which illustrated that the favorable renal function was dependent on the GPX4 [16]. Legumain could interact with GPX4 and promote lysosomal autophagy of GPX4 to promote ferroptosis in I/R-induced AKI [17]. Recently, it has been discovered that ferroptosis is involved in I/R-induced AKI and plays a significant role in renal tubular cell death [18]. Many compounds have been developed and applied in I/R-induced AKI via targeting ferroptosis. XJB-5-131 possessed a high affinity to renal tubular cells and prevented them from I/R-induced AKI via ferroptosis, not pyroptosis or necroptosis [11]. Pachymic acid could upregulate the solute carrier family 7 member A11 (SLC7A11) and GPX4 to repress ferroptosis [19]. Irisin treatment could upregulate GPX4 expression [20]. These results show that targeting ferroptosis might be a promising therapy for I/R-induced AKI.

Liproxstatin-1 (Lip-1), a spiroquinoxalinamine derivative, was discovered in 2014 [16]. It has a therapeutic effect on bronchial epithelial cell injury [21], renal fibrosis [22], pulmonary fibrosis [23], and severe acute pancreatitis-induced AKI [24]. It has been demonstrated that Lip-1 attenuated I/R-induced organ injury, such as I/R-induced hepatic damage [16], lung transplantation-induced cold I/R injury [25], and I/R-induced heart injury [26]. Nonetheless, the mechanism of Lip-1 in attenuating I/R-induced AKI is still unclear. In this study, we tried to investigate the effect and mechanism of Lip-1 in an I/R-induced AKI model and seek the key regulator. We found that Lip-1 could alleviate I/R-induced AKI through upregulating early growth response 1 (EGR1)/TP53/SLC7A11, and Lip-1 could also reduce kidney inflammation via recruiting macrophages and decreasing the release of inflammatory cytokines.

## 2. Materials and Methods

### 2.1. Mice and Renal I/R Model

The C57BL/6J mice (male, 6~8 weeks, 20~22 g) were bought from Beijing Vital River Laboratory Animal Technology Co., Ltd (Beijing, China). The mice were fostered in specific pathogen-free (SPF) cages at the Xiamen University Laboratory Animal Center. The Lip-1 was dissolved in DMSO (50 mg/mL) for storage and was adjusted to 2% DMSO (1 mg/mL) with phosphate-buffered saline (PBS) before injection. The Lip-1+I/R group was treated with Lip-1 (10 mg/kg i.p., S7699, Selleck, Houston, TX, USA) 1 h prior to I/R operation [16], while the I/R group and sham group were treated with PBS. The sham group, I/R group, and Lip-1+I/R group were anesthetized and kept on a 37 °C thermostatic table. The bilateral kidneys of the three groups were exposed via flank incision, then nontraumatic clamps were utilized to clamp bilateral renal pedicles for 30 min in the I/R group and the Lip-1+I/R group. The sham group did not experience this process. After suture, the three groups were treated with PBS by injection (1 mL, 37 °C) via i.p. and fostered in SPF cages with food and water. All the mice were sacrificed after 24 h.

### 2.2. Histological Stain and Assessment

Kidney samples were collected, washed with PBS, fixed in 4% paraformaldehyde, dehydrated, and embedded with paraffin. The paraffin blocks containing kidneys were sectioned into 5 μm thick sections and these sections were used for hematoxylin and eosin (H&E) staining, Masson staining, Periodic Acid–Schiff (PAS) staining, and Prussian Blue (PB) staining. The images were obtained by using a fluorescence microscope (Olympus IX51, Tokyo, Japan). The renal tubular injury score of H&E staining was calculated by taking a total of 50 renal tubules (5 HPFs per mouse, *n* = 6). Each renal tubular score was based on tubular epithelial cell flattening (0–1), brush border loss (0–1), cell membrane bleb formation (0–2), interstitial edema (0–1), cytoplasmic vacuolization (0–1), cell necrosis (0–2), and tubular lumen obstruction (0–2). The renal tubular injury score of Masson staining was evaluated by the total score based on each score’s signs of renal tubular damage (brush border loss, vacuolization, cell desquamation, tubule dilatation, and tubule degeneration) from 0 to 3. The renal tubular injury score of PAS staining was based on the percentages of renal tubular damage: 0, normal kidney; 1, less than 10%; 2, 10–25%; 3, 25–50%; 4, 50–75%; and 5, more than 75%.

### 2.3. Transmission Electron Microscopy (TEM)

Kidney samples were fixed with electron microscope fixative and 1% osmic acid for 2 h at room temperature, dehydrated with increasing concentrations of ethanol (30%, 50%, 70%, 80%, 95%, 100%) for 20 min per concentration and acetone twice for 15 min each time at room temperature, and then embedded with epoxy resin (90529-77-4, SPI, West Chester, PA, USA). The samples were sectioned into 60~80 nm sections with an ultramicrotome (UC7, Leica, Weztlar, Germany) and stained with 2% uranyl acetate for 8 min and 2.6% lead citrate for 8 min in a dark room. The images of tubular epithelial cells were gained via TEM (HT-7800, HITACHI, Tokyo, Japan). The mitochondrial damage score was calculated by taking a total of 50 mitochondria (5 HPFs per mouse, *n* = 6). Each mitochondrial score was based on Flameng’s scale [27]. The percentage of damage in 50 endoplasmic reticula was evaluated.

### 2.4. Renal Function

The kidney coefficient was calculated by the formula: (kidney weight × 100)/body weight. The blood of mice was collected and centrifuged at 3000 rpm to obtain serum. The creatinine (CRE) and blood urea nitrogen (BUN) were detected by a fully biochemical instrument (BS-240Vet, Mindray, Shenzhen, China) and the corresponding kit (105-000452-00 and 105-000457-00, Mindray, Shenzhen, China).

### 2.5. Renal Iron Measurement

Proper kidney tissues were homogenized in extracting solution with an iron content assay kit (BL898B, Biosharp, Hefei, China). The samples were centrifuged with 12,000× *g* at 4 °C and the supernatant solution was measured with a microplate reader (Thermo, Waltham, MA, USA) at 562 nm.

### 2.6. GSH Assay

The GSH content in renal tissues and cells were detected with a GSH assay kit (BC1175, Solarbio, Beijing, China). The renal tissues and cells were homogenized in the extracting solution of the GSH assay kit. The samples were centrifuged with 8000× *g* at 4 °C and the supernatant solution was measured with a microplate reader (Thermo, Waltham, MA, USA) at 412 nm.

### 2.7. Malondialdehyde (MDA) Assay

The MDA content in renal tissues and cells were detected with an MDA assay kit (BC0025, Solarbio, Beijing, China). The renal tissues and cells were homogenized in the extracting solution of the MDA assay kit. The samples were centrifuged with 8000× *g* at 4 °C, and the supernatant solution and reaction reagents were boiled in water for 1.5 h. The samples were centrifuged with 10,000× *g* at room temperature, and the supernatant solution was measured with a microplate reader (Thermo, Waltham, MA, USA) at 532 nm and 600 nm.

### 2.8. Collection and Bioinformatics Analysis of GEO Database

The GSE126805 (41 kidney samples after and before transplantation, post vs. pre) and GSE87487 (10 liver samples after and before transplantation, post vs. pre) databases were downloaded from the Gene Expression Omnibus (GEO) database (https://www.ncbi.nlm.nih.gov/geo/, accessed on 14 September 2022). The ferroptotic genes were downloaded from the FerrDb V2 database (http://www.zhounan.org/ferrdb/current/, accessed on 14 September 2022). The gene expression of GSE126805 and GSE87487 databases were processed with the “edgeR” package [28]. The differentially expressed genes (DEGs) between “before implantation” and “after reperfusion” in GSE126805 and GSE87487 were obtained via the “limma” package [29], and DEGs were acquired with |log2 (FC)| > 1 and *p* (adj.) < 0.05.

### 2.9. RNA Sequencing and Analysis of Mice Renal Samples

The kidneys of the sham group and the I/R group were washed with cold PBS and excluded non-kidney tissue including connective tissue and adipose tissue. Then samples contained in embedding cassettes were put into lipid nitrogen and subsequently sent to Shanghai Applied Protein Technology Co., Ltd. (Shanghai, China). for RNA sequencing to detect the mRNA expression. The DEGs were obtained through the “limma” package [29], and DEGs were acquired with |log2 (FC)| > 1 and *p* (adj.) < 0.05 (Supplementary Table S1). The GO and KEGG were performed by the “clusterProfiler” package. These results were displayed by the “ggplot2” package and the “G0plot” package [30].

### 2.10. Real Time Quantitative PCR (RT-qPCR) Assay

Total RNA of kidney tissues and HK2 cells were obtained via using the tissue RNA kit (AC0202, Shandong Sparkjade, Jinan, China) and cell RNA kit (AC0205, Shandong Sparkjade, Jinan, China), respectively, then they were reversely transcribed to cDNA by using the Evo M-MLV RT Kit (AG11711, Accurate Biotechnology, Changsha, China). The RT-qPCR was performed with the Hieff^®^qPCR SYBR Green Master Mix (11201ES08, Yeasen, Shanghai, China) and primers by using RT-PCR systems (CFX96, Bio-Rad, Hercules, CA, USA). All primers were gained from the PrimerBank (https://pga.mgh.harvard.edu/primerbank/, accessed on 31 October 2022) and synthesized by Sangon Biotech (Shanghai, China). The primer sequences are shown in Supplementary Table S2.

### 2.11. Western Blot (WB) Assay

The total protein of renal tissues and cells was extracted with the RIPA buffer (R0010, Solarbio, Beijing, China), then protein contents of samples were quantitatively detected by the Pierce™ BCA assay kit (23227, Thermo, Waltham, MA, USA). The protein samples were added into 10% SDS polyacrylamide gel for electrophoresis, transferred onto a PVDF membrane, blocked with 5% skimmed milk in a TBST solution for 1 h at room temperature, and blotted with their corresponding primary antibodies (β-actin (mouse monoclonal antibody, 1:5000, A5441, Sigma, St. Louis, MO, USA), EGR1 (rabbit monoclonal antibody, 1:1000, MA5-15008, Thermo, St. Louis, MO, USA), TP53 (mouse monoclonal antibody, 1:1000, ab26, Abcam, Cambridge, UK), SLC7A11 (rabbit monoclonal antibody, 1:1000, ab175186, Abcam, Cambridge, UK), and GPX4 (rabbit monoclonal antibody, 1:10,000, ab125066, Abcam, Cambridge, UK)) overnight at 4 °C. After washing thrice with TBST, PVDF membranes were incubated with a secondary antibody solution for 1 h at room temperature. After removing the secondary antibody solution, the PVDF membranes were detected by the Tanon 5200 system (Tanon, Shanghai, China). The densitometry of protein bands was measured by Image J.

### 2.12. Immunohistochemistry (IHC) and Immunofluorescence (IF) Assay

For the IHC assay, the paraffin sections of kidney samples were applied for 4-hydroxynonenal (4-HNE, mouse monoclonal antibody, 1:25, ab48506, Abcam, Cambridge, UK) immunohistochemistry (IHC) staining. The intensity of 4-HNE staining was detected by Image J. The paraffin sections of kidney samples were applied for F4/80 (rabbit polyclonal antibody, 1:500, GB113373, Servicebio, Wuhan, China) to observe macrophages. The images were obtained by using a fluorescence microscope (Olympus IX51, Tokyo, Japan). The counts of F4/80^+^ macrophages were counted (5 HPFs per mouse, *n* = 6). For the IF assay, the kidney tissues were fixed in a 4% paraformaldehyde solution at 4 °C, incubated in a 30% sucrose solution and then embedded in OCT (4583, Sakura, Torrance, CA, USA). The kidney samples were sectioned into 5 μm thick sections and permeabilized with a 1% Triton X-100 buffer. Then, the sections were blocked with a 10% casein solution and incubated with an EGR1 antibody solution overnight at 4 °C. In addition, the sections were incubated with IgG (H + L) antibody (A0516, Beyotime Biotechnology, Shanghai, China) for 60 min at room temperature. Finally, the sections were washed with PBS and added to DAPI (ab104139, Abcam, Cambridge, MA, USA). The images were obtained via a confocal system (FV1000MPE-B, Olympus, Tokyo, Japan).

### 2.13. Cell Line and Cell Culture

The human renal tubular cell HK2 was bought from ATCC. HK2 was cultivated in a DMEM high glucose media with 10% fetal bovine serum and cultured in a humidified incubator at 37 °C with 5% CO_2_.

### 2.14. Cell Viability

A total of 5 × 10^3^ HK2 cells were seeded into a 96-well plate and incubated overnight, then HK2 cells were managed with different concentrations of erastin (S7242, Selleck, Houston, TX, USA) and Lip-1 (1 μM) for 12 h. Then, each well was added into 10 μL MTT (5 mg/mL) and the 96-well plate was incubated for 4 h at 37 °C. Next, the medium was removed, and each well was added into 150 μL DMSO. Finally, the 96-well plate was measured with a microplate reader (Thermo, Waltham, MA, USA) at 570 nm.

### 2.15. Lentiviral Infection

The EGR1 shRNA was purchased from Shanghai Genechem. The plasmid-containing EGR1 shRNA were transfected into 293T cells with Lipofectamine 2000 for 6 h, and fresh medium was added into the 293T cell culture dish, and the viral particles were obtained from the supernatant solution after 48 h. When the density of HK2 cells reached 20–25%, the HK2 cells were infected by lentivirus for 8 h, and fresh medium was added into the HK2 cell culture dish. After the HK2 cells were cultured for 48 h, the puromycin (1.0 μg/mL) was utilized to treat cells to gain EGR1 knockdown cells.

### 2.16. Statistical Analysis

Statistical analysis (unpaired *t*-test) of data was administrated by using Prism 9. The value of *p* < 0.05 was considered significant (* *p* < 0.05, ** *p* < 0.01, *** *p* < 0.001, **** *p* < 0.0001).

## 3. Results

### 3.1. Lip-1 Alleviated I/R-Induced AKI

**Figure 1:**
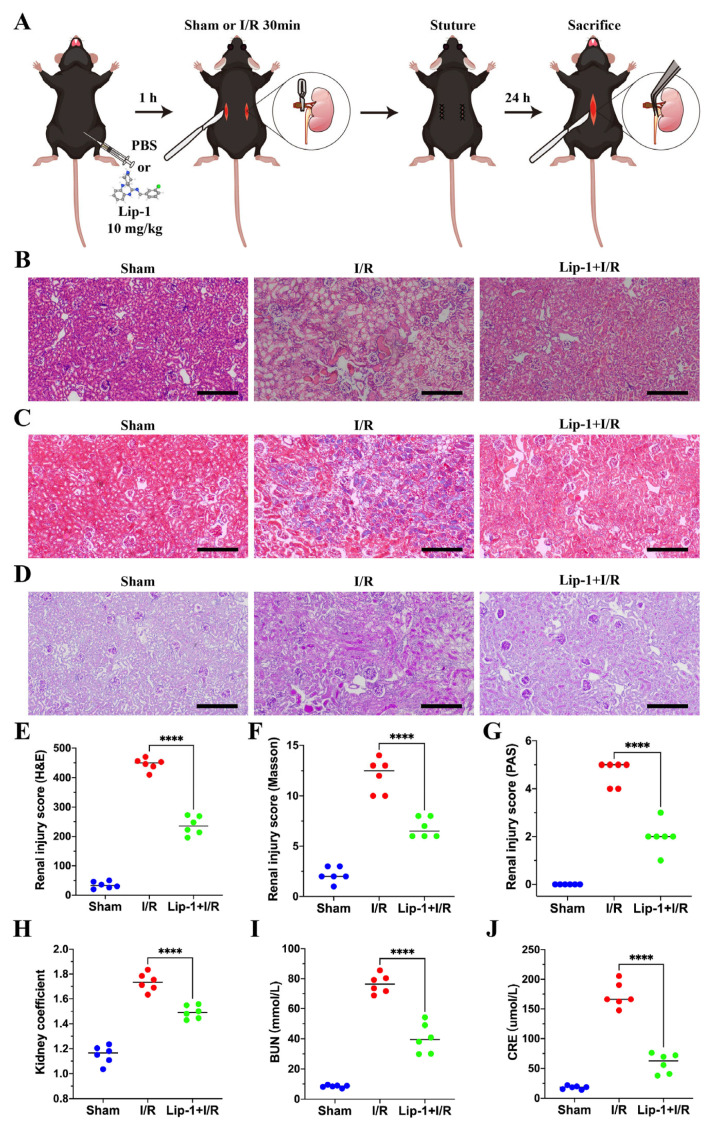
Lip-1 alleviated I/R-induced AKI. (**A**) The schema of the I/R-induced AKI model and Lip-1 management. (**B**) H&E staining of kidney, bar: 200 μm. (**C**) Masson staining of kidney, bar: 200 μm. (**D**) PAS staining of kidney, bar: 200 μm. (**E**) Renal tubular injury score of H&E staining. (**F**) Renal tubular injury score of Masson staining. (**G**) Renal tubular injury score of PAS staining. (**H**) Kidney coefficient of mice. (**I**) BUN levels of mice. (**J**) CRE levels of mice. **** *p* < 0.0001.

The administration of Lip-1 and management of I/R-induced AKI are displayed in a schematic diagram (Figure 1A). After I/R management, the renal tubular injury was evaluated in renal sections by H&E staining and it was obvious that Lip-1 could reduce renal tubular injury (Figure 1B,E). The Masson staining and PAS staining showed that vacuolization of renal tubular cells, tubular dilation, and brush border loss were observed in I/R mice, and Lip-1 could prevent the occurrence of these pathological features (Figure 1C,D,F,G). Compared with the I/R group, the kidney coefficient, BUN, and CRE significantly decreased in the Lip-1+I/R group, which indicated that Lip-1 could protect the kidney from injury (Figure 1H–J). Lip-1 could slightly reduce BUN and CRE without kidney coefficient variation (Figure S1A–C), and there was no significant tissue injury in normal mice (Figure S1D).

### 3.2. Lip-1 Suppressed Ferroptosis of Renal Tubular Cells in I/R-Induced AKI

**Figure 2:**
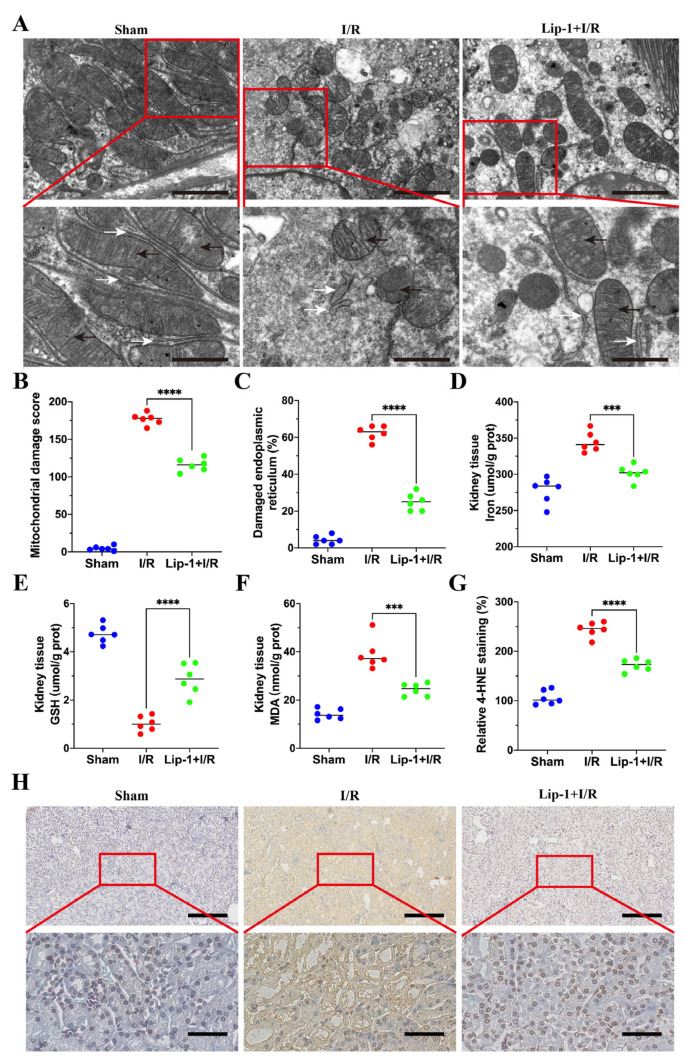
Lip-1 suppressed ferroptosis of renal tubular cells in I/R-induced AKI. (**A**) TEM images of renal tubular cells from diverse groups, bar: 2 μm (**top**) and 1 μm (**bottom**). The black arrows indicate mitochondria and the white arrows indicate endoplasmic reticula. (**B**) Quantification of mitochondrial damage in TEM of the renal tubular cells. (**C**) Quantification of damaged endoplasmic reticulum in TEM of the renal tubular cells. (**D**) Iron level of kidney. (**E**) GSH level of kidney. (**F**) MDA level of kidney. (**G**) Quantification of 4-HNE staining in kidney. (**H**) 4-HNE staining of kidney, bar: 200 μm (**top**) and 50 μm (**bottom**). *** *p* < 0.001, **** *p* < 0.0001.

According to the previous reports, ferroptosis displayed a crucial role in I/R-induced AKI [11,18,31]. The typical hallmarks of ferroptosis were observed in the renal tubular cells of the I/R group, such as increased mitochondrial membrane densities, smaller mitochondria, reduced/vanished mitochondria cristae, and swollen endoplasmic reticula, and Lip-1 treatment could reduce these changes (Figure 2A–C). The non-heme iron in the kidneys of the I/R group increased and could be reduced with Lip-1 treatment (Figure 2D). However, there was no obvious Fe^3+^ accumulation in the I/R groups with PB staining (Figure S2). Compared with the I/R group, Lip-1 increased GSH and reduced MDA in the kidney (Figure 2E,F). The IHC staining showed that the 4-HNE was remarkably increased in the I/R group, which could be apparently reversed by Lip-1 treatment (Figure 2G,H). These results suggest that Lip-1 could protect renal tubular cells from ferroptosis in I/R-induced AKI.

### 3.3. EGR1 Might Be a Crucial Regulator of Ferroptosis in I/R-Induced AKI

**Figure 3:**
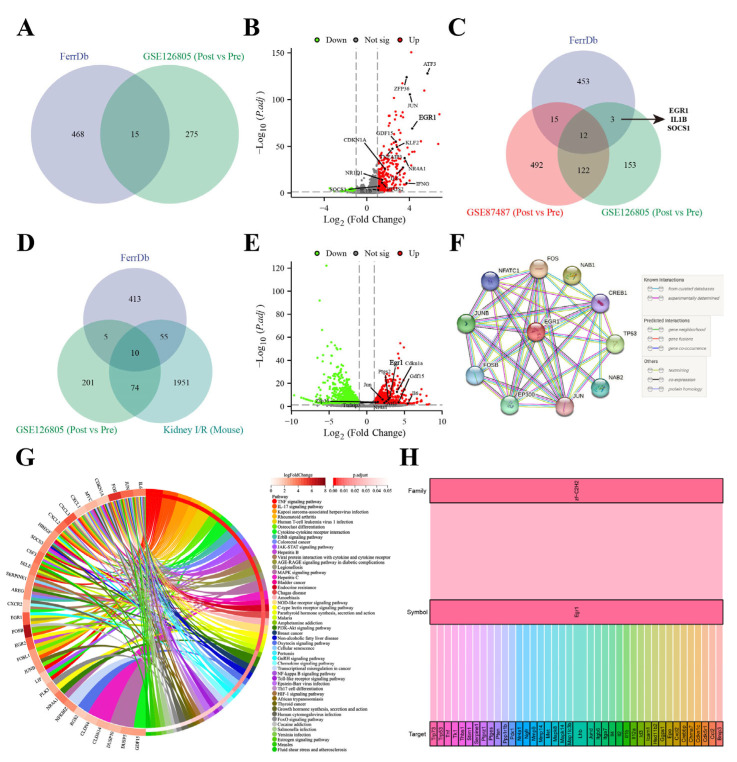
EGR1 might be a key regulator of ferroptosis in I/R-induced AKI. (**A**) Venn diagram of FerrDb and GSE126805 (post vs. pre). (**B**) Volcano plot of GSE126805 (post vs. pre). (**C**) Venn diagram of FerrDb, GSE87487 (post vs. pre), and GSE126805 (post vs. pre). (**D**) Venn diagram of FerrDb, GSE126805 (post vs. pre), and kidney samples of mouse model (I/R vs. control). (**E**) Volcano plot of kidney samples of mouse model (I/R vs. control). (**F**) The STRING interaction network of EGR1 (Homo sapiens). (**G**) Chordal graph of KEGG in GSE126805 (post vs. pre). (**H**) Sankey diagram of EGR1 and its target in I/R-induced AKI model.

A total of 290 DEGs and 641 DEGs were identified from GSE126805 (post vs. pre) and GSE87487 (post vs. pre), respectively, while 2090 DEGs were identified from kidney samples of our mouse model (I/R vs. control). There were 15 ferroptotic DEGs in the I/R of human kidneys (Figure 3A), and these DEGs are displayed in a volcano plot (Figure 3B). There were only 3 ferroptotic DEGs (EGR1, IL1B, and SOCS1) in the I/R of human kidneys (Figure 3C). There was a set of 10 ferroptotic DEGs in GSE126805 (post vs. pre) and the kidney samples of our mouse model (I/R vs. sham) (Figure 3D), and the 10 ferroptotic DEGs are displayed in a volcano plot (Figure 3E). In the chordal graph of KEGG, EGR1 might play a crucial role in GSE126805 (post vs. pre) (Figure 3G). The STRING interaction network showed that EGR1 was closely related to TP53 (Figure 3F). The Sankey diagram of RNA sequencing and analysis of mouse renal samples indicated that *Trp53* might be a target of *Egr1* in mouse models (Figure 3H). The above results indicated that EGR1 might be a crucial regulator of ferroptosis in I/R-induced AKI via regulating TP53.

### 3.4. Lip-1 Inhibited Ferroptosis of Renal Tubular Cells via Regulating EGR1/TP53/SLC7A11 in I/R-Induced AKI

**Figure 4:**
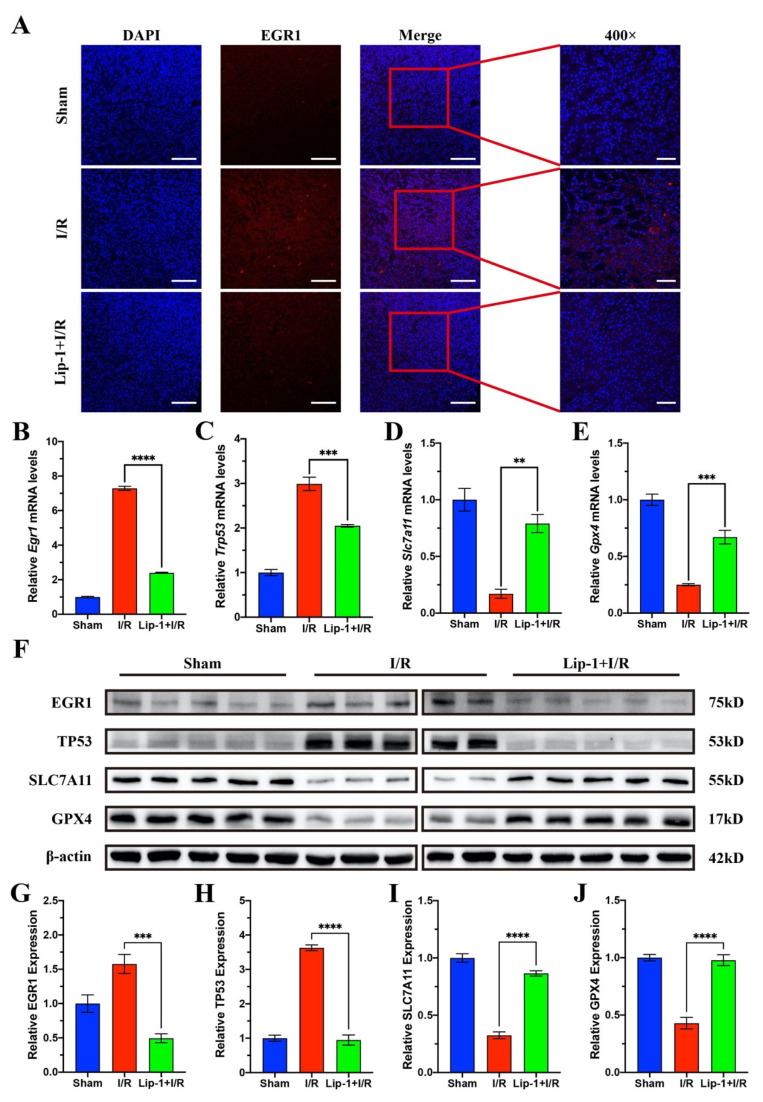
Lip-1 inhibited ferroptosis of renal tubular cells via EGR1/TP53/SLC7A11 in I/R-induced AKI. (**A**) IF of EGR1 in mouse kidney samples, bar: 50 μm. RT-qPCR results of *Egr1* (**B**), *Trp53* (**C**), *Slc7a11* (**D**), and *Gpx4* (**E**) in kidneys of mice (*n* = 5). (**F**) WB results of kidneys of mice (*n* = 5). Relative expression of EGR1 (**G**), TP53 (**H**), SLC7A11 (**I**), and GPX4 (**J**). ** *p* < 0.01, *** *p* < 0.001, **** *p* < 0.0001.

EGR1 obviously increased in the I/R group while Lip-1 treatment could significantly decrease the EGR1 expression (Figure 4A). According to previous reports, EGR1 could promote TP53 transcription [32,33]. The mRNA expression of *Egr1* in the kidneys of the I/R group increased and mRNA expression of *Trp53* was accordingly increased (Figure 4B,C). As SLC7A11 was the target of TP53 [34,35], the mRNA expression of *Slc7a11* and *Gpx4* were downregulated (Figure 4D,E). These changes were evidently reversed by Lip-1 treatment, and these results were further validated through WB (Figure 4F–J). The above results indicated that Lip-1 suppressed ferroptosis of I/R-induced AKI via regulating EGR1/TP53/SLC7A11.

### 3.5. Lip-1 Inhibited Ferroptosis of Renal Tubular Cells via Regulating EGR1 in HK2 Cells

**Figure 5:**
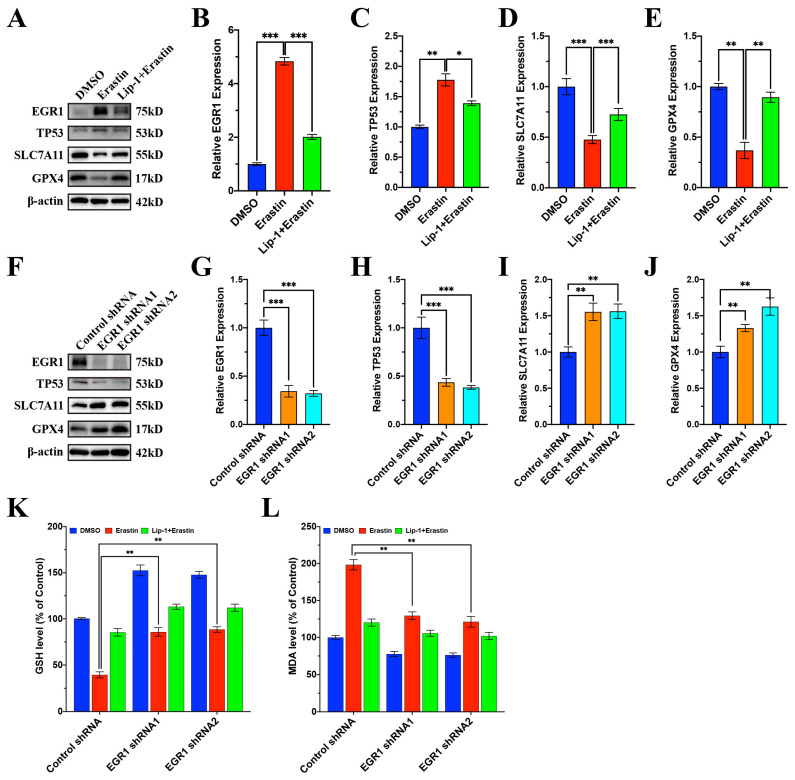
Lip-1 inhibited ferroptosis of HK2 cells via regulating EGR1. (**A**–**E**) WB assay of HK2 cell with DMSO, erastin, and Lip-1+erastin treatment. (**F**–**J**) WB assay of HK2 cell with EGR1 knockdown. (**K**) GSH levels of HK2 with DMSO, erastin, and Lip-1+erastin treatment after EGR1 knockdown. (**L**) MDA levels of HK2 with DMSO, erastin, and Lip-1+erastin treatment after EGR1 knockdown. * *p* < 0.05, ** *p* < 0.01, *** *p* < 0.001.

We used erastin to induce ferroptosis of the human tubular cell HK2 and found that 1 μM erastin could distinctly lead to HK2 cell ferroptosis, which could be reversed by Lip-1 treatment (Figure S3). The RT-qPCR results showed that *EGR1* and *TP53* were upregulated while *SLC7A11* and *GPX4* were downregulated with erastin treatment, which was reversed by Lip-1 (Figure S4). The WB results further confirmed the RT-qPCR results (Figure 5A–E). In order to investigate whether EGR1 was responsible for promoting ferroptosis, we knocked down EGR1 with shRNA; the knockdown efficiency was proven by RT-qPCR and WB (Figure S5A and Figure 5F,G). After knockdown of EGR1, the expression of TP53 was decreased while the expression of SLC7A11 and GPX4 were increased (Figure S5B–D and Figure 5F,H–J). When EGR1 was knocked down, GSH significantly increased (Figure 5K). The increased MDA in the erastin group could both be reduced in EGR1 knockdown or Lip-1 treatment (Figure 5L). The above results indicated that EGR1 was a ferroptosis promoter and Lip-1 inhibited ferroptosis via regulating EGR1 in HK2 cells.

### 3.6. Lip-1 Reduced Recruitment of Macrophages and Release of Inflammatory Cytokines In Vivo

**Figure 6:**
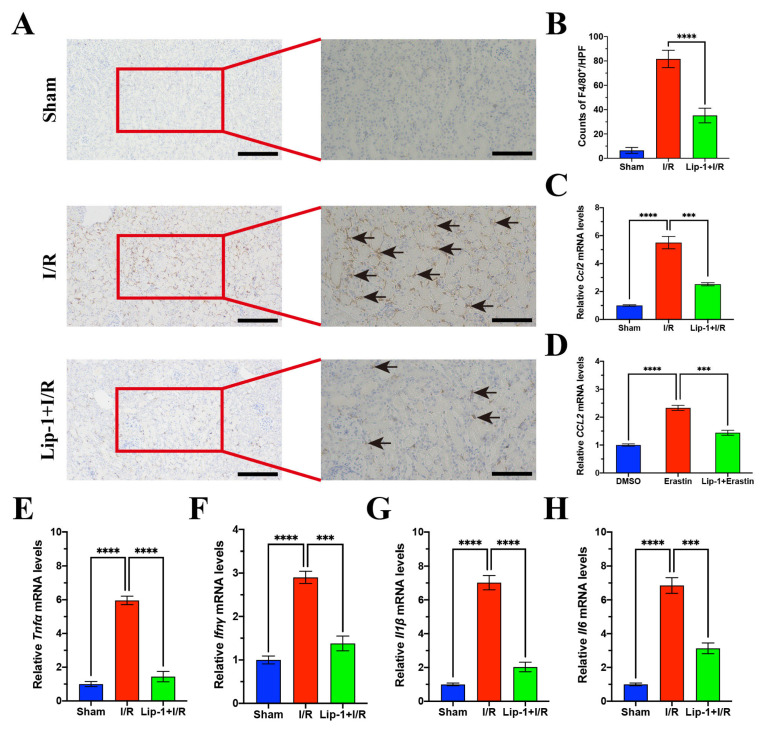
Lip-1 reduced renal inflammation. (**A**) F4/80^+^ staining of kidney samples from mice, bar: 200 μm (**left**) and 100 μm (**right**). The black arrows indicate positive F4/80^+^ staining cells. (**B**) Counts of renal F4/80^+^ macrophages in HPFs. (**C**) RT-qPCR results of *Ccl2* in kidneys of mice. (**D**) RT-qPCR results of *CCL2* in HK2 cells. RT-qPCR results of *Tnfα* (**E**), *Ifnγ* (**F**), *Il1β* (**G**), and *Il6* (**H**) in kidney samples from mice. *** *p* < 0.001, **** *p* < 0.0001.

The infiltrated F4/80^+^ macrophages in the kidneys of the I/R group markedly increased, while F4/80^+^ macrophages distinctly decreased with Lip-1 treatment (Figure 6A,B). *CCL2*, acting as a chemokine, could trigger the recruitment of F4/80^+^ macrophages. We then detected *Ccl2* expression in the kidney samples of mice, and it was discovered that Lip-1 could reduce the mRNA expression of *Ccl2* (Figure 6C). According to the previous reports [36,37], EGR1 could stimulate CCL2 transcription and secretion. Based on the Sankey diagram (Figure 3H) and the data from a previous paper [31], we found that the expression of *Ccl2* increased in the erastin group. The increased expression of *CCL2* in the erastin group of HK2 cells could be reduced by Lip-1 administration (Figure 6D). In addition, the inflammatory factors (*Tnfα*, *IFNγ*, *Il1β*, and *Il6*) significantly increased in the I/R group and these inflammatory factors decreased in the Lip-1+I/R group (Figure 6F–H). These results suggest that ferroptosis of renal tubular cells induced kidney inflammation via recruiting macrophages and releasing inflammatory cytokine, which could be reduced by Lip-1.

## 4. Discussion

AKI is a group of clinical syndromes, and it is defined as the sudden (≤7 days) and persistent decline of renal function and characterized by CRE ≥ 1.5 times baseline, an increase of ≥0.3 mg/dL within any 48 h period, or urine volume < 0.5 mL/kg for ≥6 h [38]. The one-year survival rate of patients with stage II–III AKI who resolved within 7 days is more than 90%; however, the mortality of patients in hospital who never resolved is 47% and the one-year survival rate of the remaining patients is only 77% [39]. Although the high mortality of AKI needs more attention, there is still a lack of a unified treatment plan [40]. Therefore, taking effective and protective measures in time is significant to prevent the occurrence of AKI. During renal surgery (such as renal nephrectomy), the renal vasculature needs to be blocked to ensure that the operation can be implemented (≤30 min). In addition, the donor kidney needs to be irrigated and stored in a special preservation solution with ice treatment during kidney transplantation; however, the organ preservation solution usually does not contain a protective agent. Hence, investigating the mechanism of I/R-induced AKI and developing effective drugs are urgent for medical researchers.

Ferroptosis could be summarized by the following characteristics: metabolic disorder of iron, destruction of cellular antioxidant system, production of reactive oxygen species, and accumulation of lipid peroxides [41]. Ferroptosis has been regarded as the potential target to prevent or cure AKI. It was found that ferroptosis might be more correlative than necroptosis and apoptosis in I/R-induced AKI [18,42]. Erastin is the first compound that could trigger ferroptosis via inhibiting system X_c_^−^, which could suppress GSH synthesis [43]. GPX4 transforms lipid peroxides to corresponding alcohols, and GSH could reduce the oxidated active site selenol of GPX4 [44]. The erastin-induced ferroptosis could be prevented by many compounds, such α-tocopherol, ferrostatin-1, and quercetin [31,45]. Lip-1 was screened from over 40,000 drug-like small molecules, and it could repress buthionine sulfoximine-, erastin-, and RSL3-induced ferroptosis of mouse embryonic fibroblast cells at low concentrations [16]. In our study, Lip-1 could significantly protect kidneys from I/R-induced AKI, however its application is limited because it should be assisted with DMSO and a cosolvent (such as polyethylene glycol) to enhance solubility. The absorption and pharmacokinetics of Lip-1 need be further investigated. We used 10 mg/kg Lip-1 before renal I/R and the effect on kidney protection was obvious, and indicated that Lip-1 indeed arrived in renal tubular cells and played a role in protecting them from ferroptosis in renal I/R. Although poor solubility restricted the application of Lip-1, optimizing the chemical structure of Lip-1 might make it obtain higher bioavailability and solubility.

Ferroptosis is a novel regulable cell death. Although the iron level was significantly increased in the kidney samples of the I/R group, the PB staining of kidneys in I/R showed no iron content. The results might be attributed to the iron content assay kit that mainly detected the Fe^2+^ (not Fe^3+^), thus it could be speculated that the increased iron content in kidneys of I/R was mainly Fe^2+^. According to the results of RNA sequencing and analysis, we identified EGR1 as the key molecule that promotes ferroptosis. EGR1 is a zin-finger transcription factor of the immediate early gene family [46], and it could be rapidly induced by hypoxia, growth factors, and other regents [47]. EGR1 could bind to the promoter regions of many downstream target genes via its three DNA-binding domains [48]. The expression of EGR1 could not be detected in normal adult kidneys [49], and it regulates inflammation and fibrosis in various tissues including kidneys [46]. Recent research showed that EGR1 played a renoprotective effect via increasing SOX9 expression by directly binding to the promoter of the *Sox9* after I/R- and folic acid-induced AKI [50]. In another research study, EGR1 was regarded as a ferroptosis inducer in acute myocardial infarction because it inhibited GPX4 to promote ferroptosis via miR-15a-5p [51]. In our investigation, we firstly demonstrated that EGR1 was a ferroptosis inducer in I/R-induced AKI. Knockdown of EGR1 could significantly increase expression of SLC7A11 and GPX4 via downregulating TP53 expression, accompanied by an elevation of GSH and a reduction of lipid peroxides. We also found that Lip-1 could distinctly decrease EGR1 expression during I/R-induced AKI, which demonstrated that Lip-1 inhibited ferroptosis through suppressing EGR1 expression.

It has been proven that several types of cell death participate in I/R-induced AKI, however there is still no consensus on which type of cell death plays the core role in I/R-induced AKI. Ferroptosis plays a significant role in I/R-induced AKI and could lead to inflammation because ferroptotic renal tubular cells could release CCL2 and recruit macrophages. According to the RNA-sequence analysis, EGR1 was involved in ferroptotic renal tubular cells. EGR1 could promote inflammation in various diseases, such as cholestatic liver, I/R-induced lung injury, and atherogenesis [52,53,54]. *Egr-1* deficiency in primary renal tubuloepithelial cells isolated from mice could weakly respond to pro-inflammatory and pro-fibrotic stimuli ex vivo, and *Egr1*^−/−^ mice with tubulointerstitial nephritis expressed less TNFα and CCL2 [46]. Lip-1 could apparently reduce the expression of EGR1 and CCL2, reduce the infiltration of macrophages in vivo, and reduce levels of inflammatory cytokines in renal tubular cells.

## 5. Conclusions

**Figure 7:**
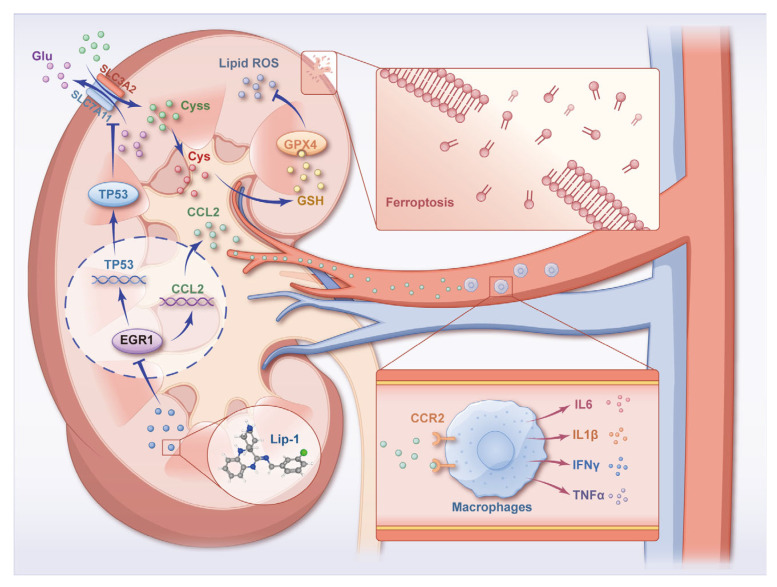
Schema of the mechanism of Liproxstatin-1 on renal ischemia/reperfusion.

In summary, our research proved that Lip-1 could alleviate I/R-induced AKI via suppressing ferroptosis (Figure 7). Our investigation indicated that (i) Lip-1 suppressed ferroptosis of renal tubular cell in I/R-induced AKI; (ii) EGR1 might be a key regulator of ferroptosis in I/R-induced AKI; (iii) Lip-1 inhibited ferroptosis of renal tubular cell via regulating EGR1/TP53/SLC7A11; and (iv) Lip-1 reduced infiltration of macrophages and release of inflammatory cytokines. This work paves the theoretical basis of new therapeutic strategies for I/R-induced AKI.

## Data Availability

The data presented in this study are available on reasonable request from the corresponding author.

## Supplementary Materials

The following supporting information can be downloaded at: https://www.mdpi.com/article/10.3390/antiox13020182/s1. Table S1: All genes of I/R vs control assayed by RNA-sequence. Table S2: Primer sequences used for RT-qPCR. Figure S1: Management with Lip-1 and corresponding solvent in vivo. (**A**) Kidney coefficient of mice. (**B**) BUN levels of mice. (**C**) CRE levels of mice. (**D**) H&E staining of heart, liver, spleen, lung, and kidney (*n* = 5), bar: 200 μm. Figure S2: PB staining of kidney from diverse groups, bar: 200 μm. Figure S3: Various concentrations of erastin and Lip-1 (1 μM) were used to treat HK2 cells. Figure S4: RT-qPCR results of *EGR1* (**A**), *TP53* (**B**), *SLC7A11* (**C**), and *GPX4* (**D**) in HK2 with DMSO, erastin, and Lip-1+erastin treatment. Figure S5: RT-qPCR results of *EGR1* (**A**), *TP53* (**B**), *SLC7A11* (**C**), and *GPX4* (**D**) in HK2 with EGR1 knockdown.
