# Supplementary material for: Liproxstatin-1 Alleviated Ischemia/Reperfusion-Induced Acute Kidney Injury via Inhibiting Ferroptosis
Source: Antioxidants (Basel). 2024 Jan 31;13(2):182. doi: 10.3390/antiox13020182 (PMC10886111; doi:10.3390/antiox13020182)
Supplement: Supplementary file 1 [file antioxidants-13-00182-s001.zip › Supplementary Table S2.pdf]

Supplementary Table S2 Primer sequences used for RT-qPCR.

Species	Gene		Sequence (5'to 3')
Mouse	β-actin	F	GGCTGTATTCCCCTCCATCG
		R	CCAGTTGGTAACAATGCCATGT
	Egr1	F	TCGGCTCCTTTCCTCACTCA
		R	CTCATAGGGTTGTTCGCTCGG
	Trp53	F	GTCACAGCACATGACGGAGG
		R	TCTTCCAGATGCTCGGGATAC
	Slc7a11	F	GGCACCGTCATCGGATCAG
		R	CTCCACAGGCAGACCAGAAAA
	Gpx4	F	GCCTGGATAAGTACAGGGGTT
		R	CATGCAGATCGACTAGCTGAG
	Ccl2	F	TTAAAAACCTGGATCGGAACCAA
		R	GCATTAGCTTCAGATTTACGGGT
	Tnfα	F	GACGTGGAACTGGCAGAAGAG
		R	TTGGTGGTTTGTGAGTGTGAG
	Ifnγ	F	ATGAACGCTACACACTGCATC
		R	CCATCCTTTTGCCAGTTCCTC
	Il1β	F	GCAACTGTTCCTGAACTCAACT
		R	ATCTTTTGGGGTCCGTCAACT
	Il6	F	CCAAGAGGTGAGTGCTTCCC
		R	CTGTTGTTCAGACTCTCTCCCT
Homo	β-actin	F	CGTGCGTGACATTAAGGAGAAG
		R	GGAAGGAAGGCTGGAAGAGTG
	EGR1	F	CTGCGACATCTGTGGAAGAAA
		R	TGTCTGCTTTCTTGTCCTTCTG
	TP53	F	CCCAAGCAATGGATGATTTGA
		R	GGCATTCTGGGAGCTTCATCT
	SLC7A11	F	TCTCCAAAGGAGGTTACCTGC
		R	AGACTCCCCTCAGTAAAGTGAC
	GPX4	F	TGGGAAATGCCATCAAGTGG
		R	GGTCCTTCTCTATCACCAGGGG
	CCL2	F	CAGCCAGATGCAATCAATGCC
		R	TGGAATCCTGAACCCACTTCT
