# Supplementary figures and images for: Liproxstatin-1 Alleviated Ischemia/Reperfusion-Induced Acute Kidney Injury via Inhibiting Ferroptosis
Source: Antioxidants (Basel). 2024 Jan 31;13(2):182. doi: 10.3390/antiox13020182 (PMC10886111; doi:10.3390/antiox13020182)

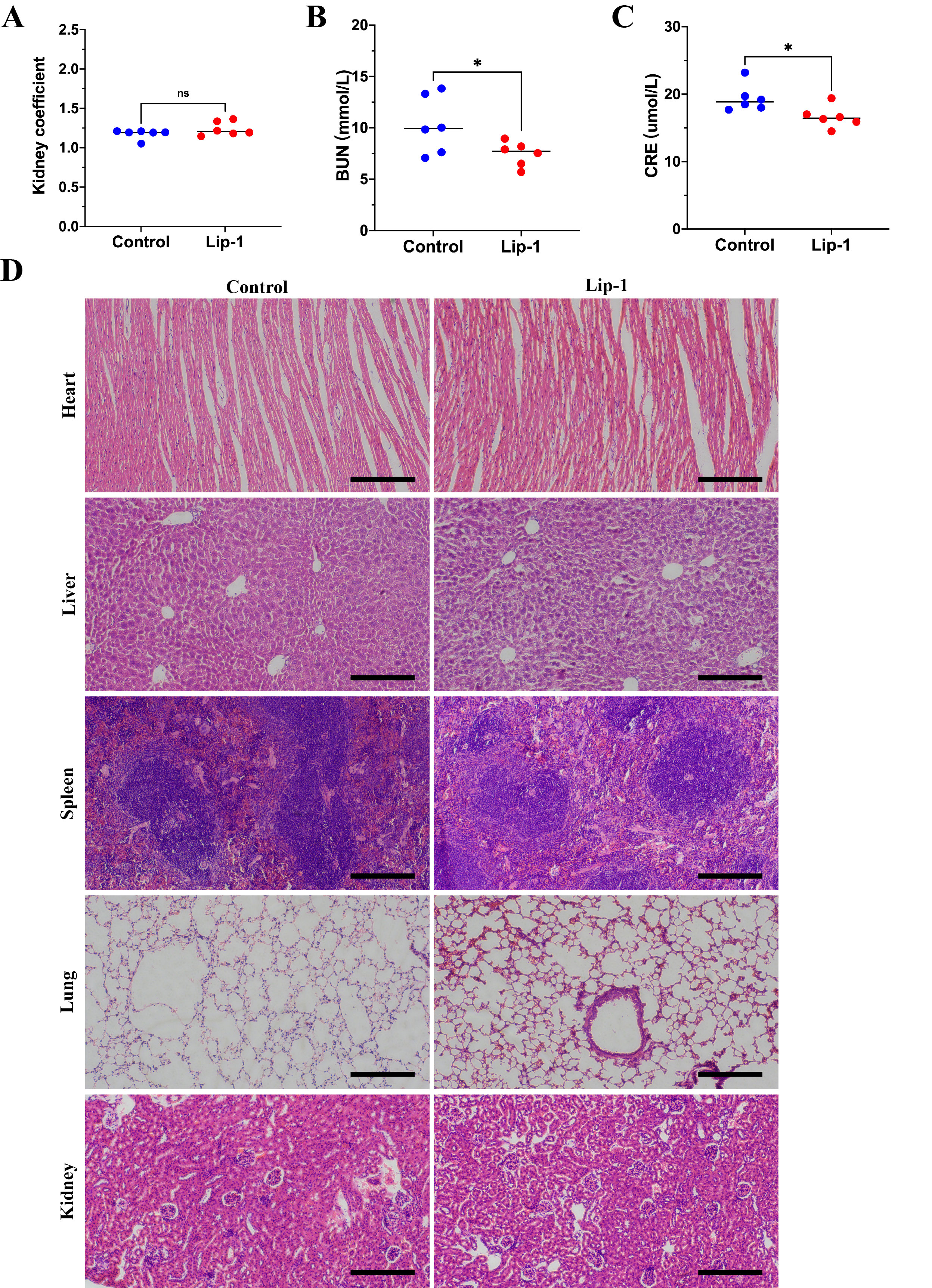

Supplement: Supplementary file 1 [file antioxidants-13-00182-s001.zip › Supplementary Figure S1.jpg]

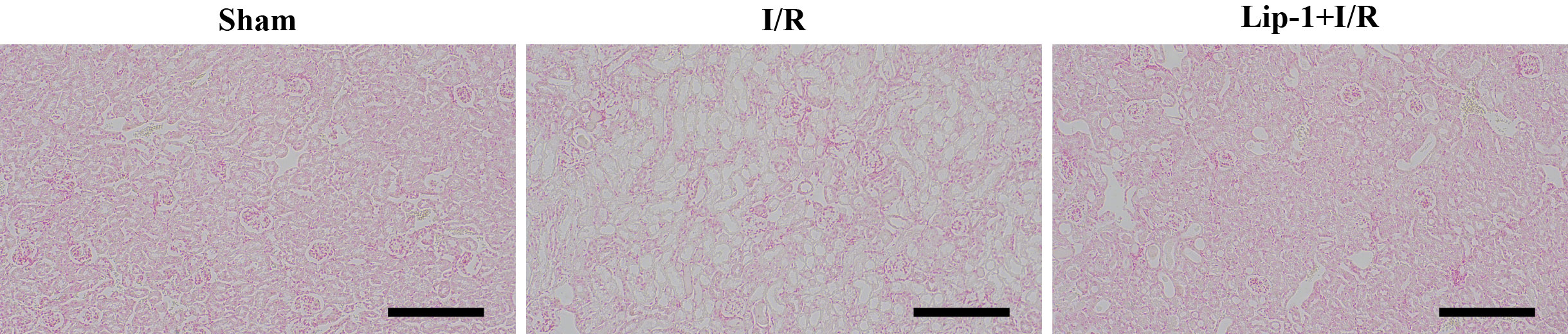

Supplement: Supplementary file 1 [file antioxidants-13-00182-s001.zip › Supplementary Figure S2.jpg]

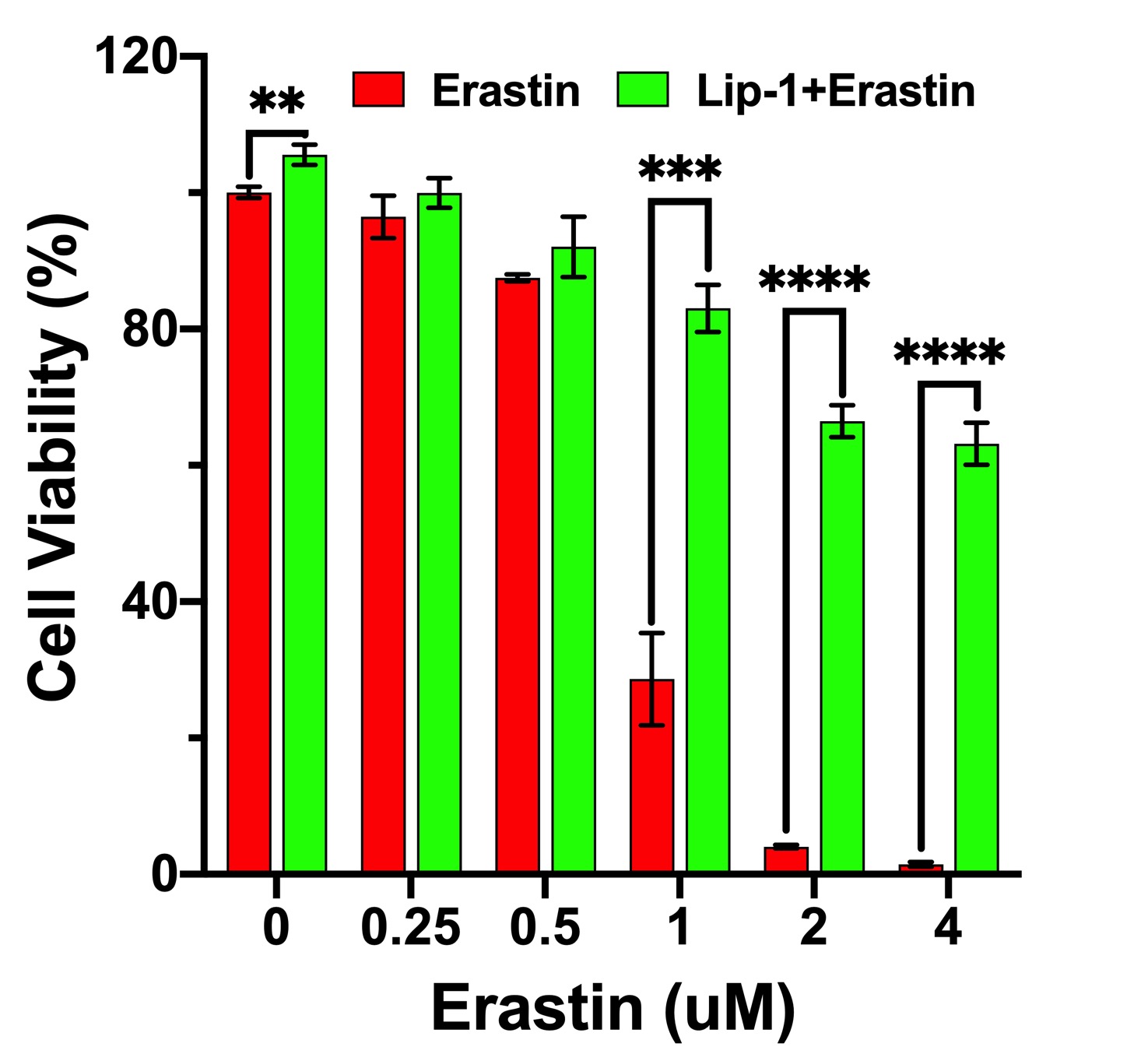

Supplement: Supplementary file 1 [file antioxidants-13-00182-s001.zip › Supplementary Figure S3.jpg]

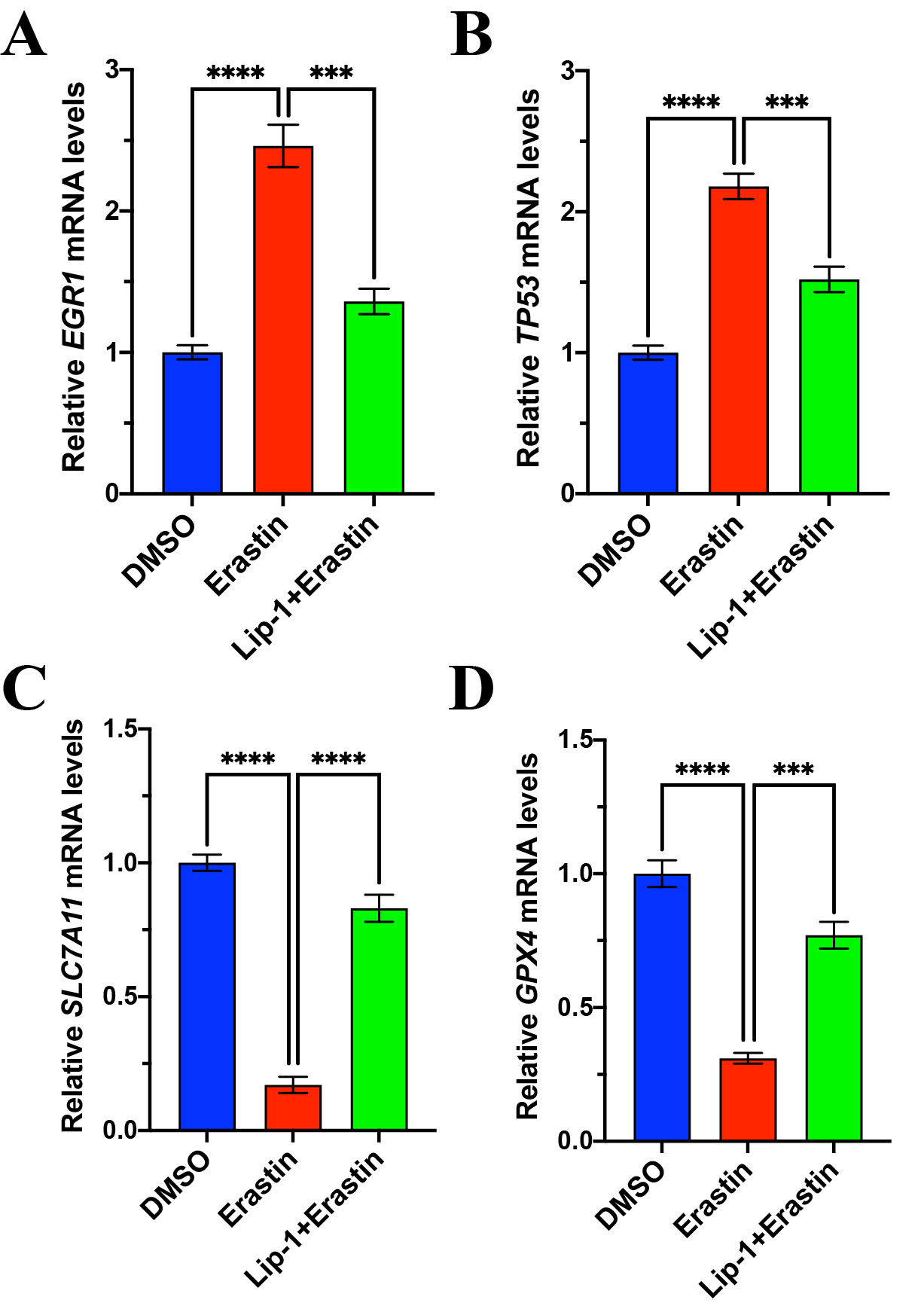

Supplement: Supplementary file 1 [file antioxidants-13-00182-s001.zip › Supplementary Figure S4.jpg]

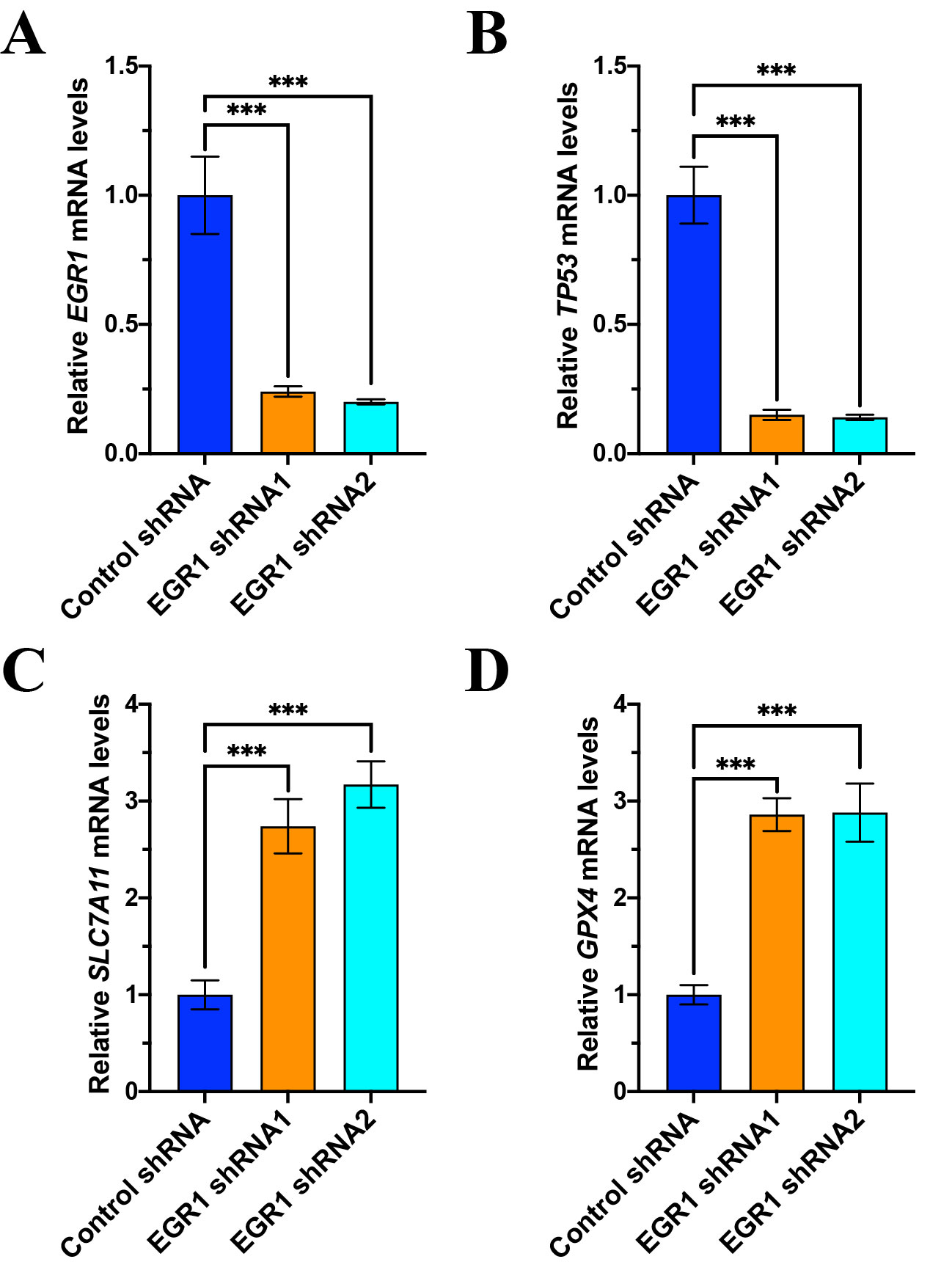

Supplement: Supplementary file 1 [file antioxidants-13-00182-s001.zip › Supplementary Figure S5.jpg]
